# Physical activity-related indicators in children and adolescents in Uruguay: A scoping review based on the Global Matrix initiative

**DOI:** 10.3389/fpubh.2022.954621

**Published:** 2022-09-07

**Authors:** Bruno Bizzozero-Peroni, Sofía Fernández-Giménez, Enrique Pintos-Toledo, César Augusto Corvos, Valentina Díaz-Goñi, Javier Brazo-Sayavera

**Affiliations:** ^1^Instituto Superior de Educación Física, Universidad de la República, Rivera, Uruguay; ^2^Grupo de Investigación en Análisis del Rendimiento Humano, Universidad de la República, Rivera, Uruguay; ^3^Health and Social Research Center, Universidad de Castilla-La Mancha, Cuenca, Spain; ^4^Instituto Superior de Educación Física, Universidad de la República, Maldonado, Uruguay; ^5^PDU EFISAL, Centro Universitario Regional Noreste, Universidad de la República, Rivera, Uruguay; ^6^Department of Sports and Computer Science, Universidad Pablo de Olavide, Seville, Spain

**Keywords:** evidence synthesis, health behaviors, physical activity surveillance, health promotion, youth

## Abstract

**Background:**

The first Uruguay's Report Card in 2018 based on the Global Matrix initiative showed the lack of information on physical activity in children and adolescents. This study mapped and examined the available evidence on physical activity-related indicators based on Uruguay's 2022 Report Card.

**Methods:**

The scoping review was reported using the Joanna Briggs Institute and the Preferred Reporting Items for Systematic Reviews extension for Scoping Reviews guidelines. A comprehensive literature search was performed for the period between 2018 and 2021, including electronic databases (PubMed, Web of Science, LILACS, Scielo, and Latindex), gray literature (Google Scholar, open access thesis, relevant websites of State-agencies and International Organizations), national and regional relevant journals, and reference lists of key texts. Two researchers independently conducted both the selection and data-charting process. Data items from each paper were charted based on the Population, Concept, and Context elements reflected in the objective of the review. A narrative synthesis and network plots were conducted to summarize the evidence.

**Results:**

A total of 20 papers were included in this review, consisting of four peer-reviewed scientific papers, three bachelor's theses, four official documents of State-agencies, four Government reports, of which three included national surveys, and five laws. Strengths, weaknesses, and knowledge gaps were identified from the available evidence. We synthesized main challenges such as publishing scientific studies, establishing cross-national and cross-sectoral collaborations in research projects, generating high-quality data, reporting information on social inequality indicators that influence equitable distribution, or increasing access to public information. Our results support early emerging and growth research on this topic. However, despite existing papers on physical activity-related indicators in Uruguayan youths, the lack of high-quality evidence remains clear.

**Conclusion:**

The findings of this scoping review provide the best available evidence for identifying and overcoming the challenges of physical activity-related indicators research in Uruguay. The methodological framework used could be useful for countries involved in future editions of the Global Matrix initiative.

**Systematic review registration:**

Open Science Framework, https://osf.io/hstbd/.

## Introduction

Physical activity (PA) is a fundamental pillar in the health and well-being of children and adolescents (1, 2). In contrast, insufficient PA is one of the major modifiable risk factors for mortality and non-communicable diseases (NCDs) worldwide (3, 4), with an estimated increased risk of death of 20–30% compared with physically active individuals (5). Globally, low levels of PA are causing growing health alarms (6). Specifically, among children and adolescents, 81% worldwide do not meet PA recommendations (7, 8). In line with this global pandemic (9), only 13.8% of Uruguayan adolescents aged 13–17 years were physically active at least 60 min daily (10). Consequently, this calls for urgent policy strategies to increase young population PA levels and reduce the burden of NCDs and the health-related consequences (7).

One of the strategic actions recognized by the World Health Organization (WHO) for this challenge is the continuous improvement of nationwide data systems that support regular PA surveillance (11). In this context, the Global Matrix (GM) initiative, led by the Active Healthy Kids Global Alliance since 2014, has arisen for the need to monitor the PA levels in youth nationally and globally (12). For this purpose, a national Report Card was developed for each participating country on PA-related indicators linked to daily behaviors (physical activity, sports participation, active play, active transportation, sedentary behavior, and physical fitness) and contexts and sources of influence (family and peers, school environment, community and environment, and government) (12). This knowledge translation tool allows to grade these indicators, synthesize the available evidence, and identify research gaps (13).

In addition to the low-average grade of the PA-related indicators in Uruguayan children and adolescents (14), the first Uruguay's Report Card in 2018 showed a limited number of papers (*n* = 7) to draw consistent conclusions, and the lack of information in this field with three (active play, family and peers, and community and environment) out of 10 indicators with insufficient information (15). In a general Latin American context with limited research capacity on the PA-related indicators (16, 17), Uruguay is facing important research challenges. The lack of reliable data to allow epidemiological characterization is one of the main weaknesses in national research (18). Uruguay's 2022 Report Card (19) reflects a major effort in this regard. To complement this approach, this study allows for a more detailed breakdown of the evidence by identifying strengths, weaknesses, and research gaps. Therefore, this scoping review mapped the literature on PA-related indicators based on the GM initiative in Uruguayan children and adolescents.

## Methods

A scoping review (ScR) was conducted using Arksey and O'Malley's five-stage framework (stages 1 to 5: identifying the research question, identifying relevant studies, study selection, charting the data, and collating, summarizing, and reporting the results) (20) and the Joanna Briggs Institute guidelines (21). The ScR was reported by the Preferred Reporting Items for Systematic Reviews and Meta-Analyses extension for Scoping Reviews checklist (22) (Supplementary Table S1). The protocol of this ScR was registered with the Open Science Framework (23).

An ScR allows for a broad and comprehensive review of the existing literature, and to identify research gaps (21). Therefore, we used this type of review to provide a “map” of the available evidence for our research questions based on the Population, Concept, and Context (PCC) components (21): “Which evidence is available for PA-related indicators in Uruguayan children and adolescents?” and “Are there strengths, weaknesses, or evidence gaps identified for PA-related indicators in Uruguayan children and adolescents?” Additionally, the following sub-question was addressed: “What are the PA-related indicators and age ranges of the Uruguayan children and adolescents where evidence is available?”.

### Eligibility criteria

For the purposes of this ScR, the papers referred to all types of documents, such as scientific publications, government documents or reports, open access theses, and laws. To be included, papers needed to focus on the PCC elements:

– Population: Uruguayan children (5–12 years) and adolescents (13–17 years).
– Concept: PA-related indicators of the GM initiative linked to daily behaviors [overall physical activity (PA), organized sport and PA participation (SP), active play (AP), active transportation (AT), sedentary behavior (SB), and physical fitness (PF)], settings and sources of influence [Family and Peers (FAM), school environment (SCH), and Community and Environment (COM)], and strategies and investments [government (GOV)].
– Context: Uruguay.

We included papers that reported quantitative information on the benchmarks of PA-related indicators (24) in Uruguayan children and adolescents. No restrictions were applied based on the type of paper (e.g., observational studies, randomized controlled trials, gray literature, governmental report), only for qualitative reports. Additionally, we excluded papers that do not fit into the conceptual framework of the ScR, focused on a communicable chronic condition or in a specific interest group (e.g., functional disabilities and substance abuse).

### Search strategy and information sources

To identify potentially relevant documents, a comprehensive literature search was performed using two methods.

First, a systematic search of peer-reviewed journal papers was conducted on MEDLINE (PubMed), Web of Science, LILACS, Scielo, and Dialnet (Latindex) from 01/012018 up to 31/12/2021. The search strategy was performed using the following terms based on the PCC mnemonic (21): [Population: (“children” OR “adolescent”)] AND [Concept: (“physical activity” OR “sedentary” OR “active commuting” OR “outdoor play” OR “fitness” OR “sport” OR “policy” OR “built environment” OR “school” OR “family”)] AND [Context: (“Uruguay”)]. Furthermore, manual searches in reference lists of retrieved documents and both national and regional relevant journals were performed to identify potential papers for inclusion. The complete and detailed search strategies for each database are provided in Supplementary Table S2.

Second, gray literature identified *via* Google Scholar (using the main terms based on the PCC mnemonics detailed above), open access thesis (BiUR database, University of the Republic), and relevant websites of State-agencies (Presidency, Ministries, Regional Governments, or Municipalities, National Government Agencies, Autonomous State Entities, and Decentralized State Services) and International Organizations (WHO, Pan American Health Organization) were searched for the identification of papers *via* other methods.

All searches were performed between 2018 and 2021, based on the 4-year update of the literature available for the GM 4.0 project's PA-related indicators gradings reporting (12). Even so, documents published before 2018 were also included in this study because they refer to laws or educational guidelines that remain in force and are crucial for the analysis of Uruguayan children and adolescents' PA-related indicators. Additionally, a longer search date was established than the one used in Uruguay's 2022 Report Card methodology (19) to include more papers that would allow for a broader answer to the research questions. Therefore, Uruguay's 2022 Report Card was based on the evidence included in this ScR, except for papers published after the study search date implemented for the Report Card (19).

### Selection process

The screening process was performed by ordering the references using the Mendeley reference management software (version 1.19.8) according to the inclusion/exclusion criteria. An Excel standardized table (v.11) was used for the selection and extraction process to establish agreement among reviewers.

First, two independent reviewers (BBP and JBS) screened the titles and abstracts of documents for potential inclusion. In cases where a decision for exclusion or potential inclusion cannot be made, the full text was retrieved. Second, two independent reviewers (BBP and JBS) decided on the inclusion or exclusion of the full-text documents based on the selected criteria by completing a checklist form. We resolved disagreements on study selection by consensus with all reviewers if needed.

### Data items and data charting process

Two reviewers (BBP and JBS) developed an Excel (v.11) standardized table to chart the data from specific domains of the PCC elements (Table 1). The following data items from each paper were charted and collated independently by two researchers (BBP and JBS): authors and year of publication; paper design; participant's characteristics (sample, age, % male/female), PA-related indicators (Supplementary Table S3), PA-related indicators' findings according to the gender sub-analysis, and summary from available evidence (Supplementary Table S4). Disagreements on data extraction were consensual between the reviewers, who continuously updated the data-charting form in an iterative process. The synthesis of results was presented through the data-charting form (Supplementary Table S4). Disagreements on data extraction were discussed and resolved by two reviewers (BBP and JBS), who continuously updated the data-charting form in an iterative process. Besides, a descriptive summary of the main results for the available evidence on the PA-related indicators in Uruguayan children and adolescents was presented. Moreover, we extracted a series of network geometry graphs from the coded synthesized data to show several associations between PA-related indicators, age, gender, type of evidence, and key findings from evidence (strengths, weaknesses, and research gaps). All this information was used to identify topics based on the research questions to create the narrative. Network geometry plots were performed using the STATA SE software, version 15 (StataCorp, College Station, TX, USA).

**Table 1:** Data collection domains for data extraction and charting.

**Domain**	**Details**
Population	Description of the age ranges of Uruguayan children and adolescents where evidence is available for PA-related indicators.
Concept	Description of evidence available on PA-related indicators of the GM initiative. Description of strengths, weaknesses, and gaps in the available evidence of PA-related indicators in Uruguayan children and adolescents.
Context	Description of evidence available on national levels. Any mention of specific issues (e.g., gender, socioeconomic status) that influenced the Uruguayan children and adolescents' PA-related indicators.

GM, Global Matrix; PA, overall physical activity.

### Dealing with missing data

We contacted one paper's authors to access additional relevant material (i.e., missing PA-related indicators outcome). Springer et al. (25) sent the Supplementary material.

### Critical appraisal

According to the guidelines for ScR, no quality assessment is required (21, 22) and, therefore, an overview of the existing evidence was achieved regardless of methodological quality or risk of bias.

## Results

### Selection of sources evidence

**Figure 1:**
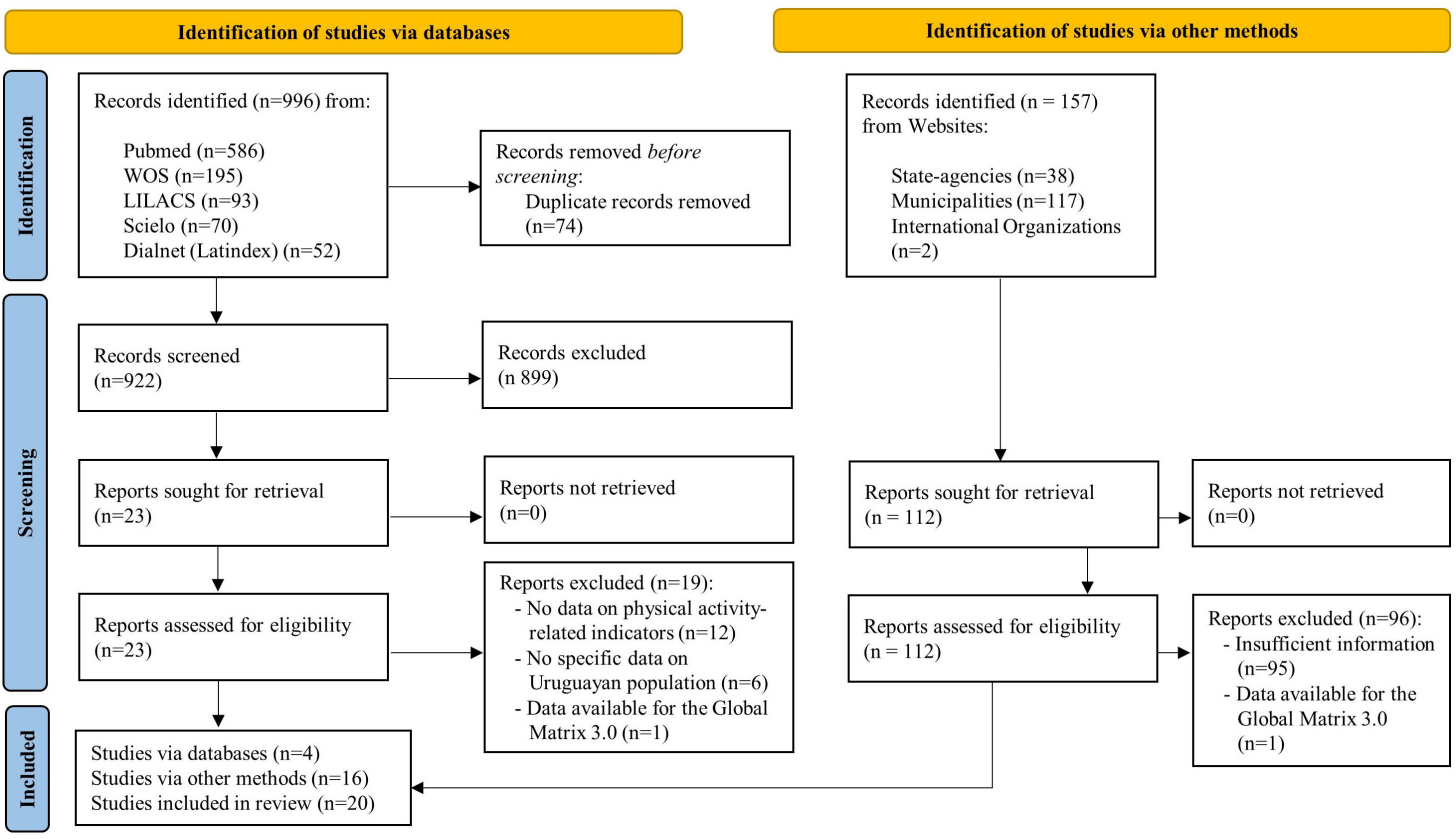
PRISMA flow diagram for identifying, screening, and determining eligibility and inclusion of papers.

A total of 1,153 records were assessed, comprising 996 studies from databases (PubMed, WOS, LILACS, Scielo, and Latindex) and 157 reports from websites (State-agencies, Regional Governments, Municipalities, and International Organizations). Finally, 20 papers were included in this ScR (Figure 1).

### Study characteristics

#### Study design

Supplementary Table S3 shows the main characteristics of the included papers. Among the 20 papers included in the ScR, five were current laws of the Uruguayan Parliament (26–30), four were peer-reviewed scientific papers (25, 31–33), government reports (10, 34–36), or official documents of a State educational agency (37–40), and three were open access bachelor's thesis (41–43).

Of the peer-reviewed scientific papers, three were cross-sectional (31–33) and one was a randomized controlled trial (25). Of the open access bachelor's thesis, the three papers were cross-sectional (41–43). Furthermore, three government reports included national surveys (10, 34, 35) and one official document from the National Agency of Education were reported together with a government entity (37).

#### Year of publication

The papers were conducted between 2018 and 2021, except for one official document of a State educational agency (39) and three laws (26, 29, 30) that were published before 2018. These documents are still in force and are crucial for the analysis of PA-related indicators of Uruguayan children and adolescents, therefore, were included.

#### Population

**Figure 2:**
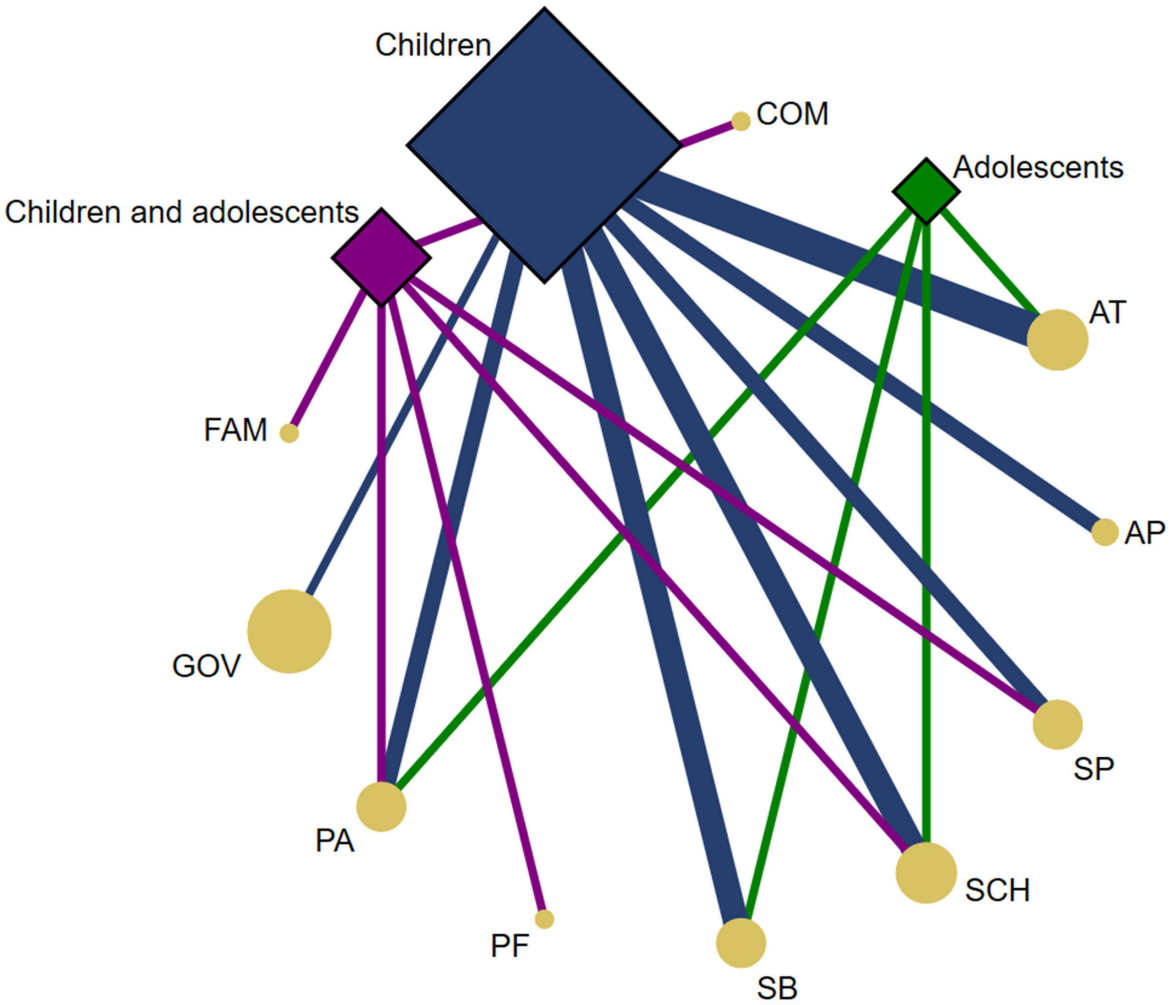
Network geometry plots of available comparisons between the PA-related indicators and the age groups. The size of the circular nodes (PA-related indicators) was relative to the number of papers analyzing these components. The size of the diamond nodes (age groups) was relative to the number of available data on PA-related indicators analyzing these components. The width of the solid line connecting the nodes was relative to the number of papers analyzing the PA-related indicators (circular nodes) according to age groups (diamond nodes). AP, Active play; AT, Active transportation; COM, Community and built environment; FAM, Family and Peers; GOV, Government; PA, Physical activity; PF, Physical fitness; SB, Sedentary behavior; SCH, School environment; SP, Sport and physical activity participation.

Data samples were collected between 2015 and 2020. Of the papers included (12 out of 20) involving children and adolescents, the range of sample sizes was 55–136,483 (10, 25, 31–35, 37, 38, 41–43). The age range of the subjects included in these papers was 5–17 years, except for two papers that analyzed the PA-related indicators also in the adult population (34, 35). Five papers reported data on children and adolescents aged 9–17 years (25, 31, 34, 35, 37), four papers analyzed children aged 6–12 years (38, 41–43), two focused on children aged 5–6 years old (32, 33), and one paper focused specifically on adolescents aged 13–17 years (10). The PA-related indicators analyzed by age groups are detailed in Figure 2.

#### Socioecological setting

The most common socioecological setting targeted was the Primary school in 10 papers (30–33, 37–39, 41–43), followed by five papers that focused on the sports system (27–29, 34, 36). Moreover, two papers referred to secondary education institutions (25, 40) or targeted the general population without addressing a specific institutional framework (10, 35), and one paper focused on the general education environment (26).

### Synthesis of findings

Findings from the included papers are synthesized in Supplementary Table S4, in the network's geometry graphs, and in the following sections.

### Study purpose

The main purpose of the papers included was PA-related indicators, except for two laws that focused on a broader topic such as the education system (26, 27), two other laws that focused on the sports system in general (28, 29), and one government report that referred to different broad domains of adolescents' lives such as education and health (35). Finally, the scientific papers reported updated data on the PA-related indicators based on the analysis of obesity, cardiovascular system, and healthy habits (25, 31–33).

### Physical activity indicators

**Figure 3:**
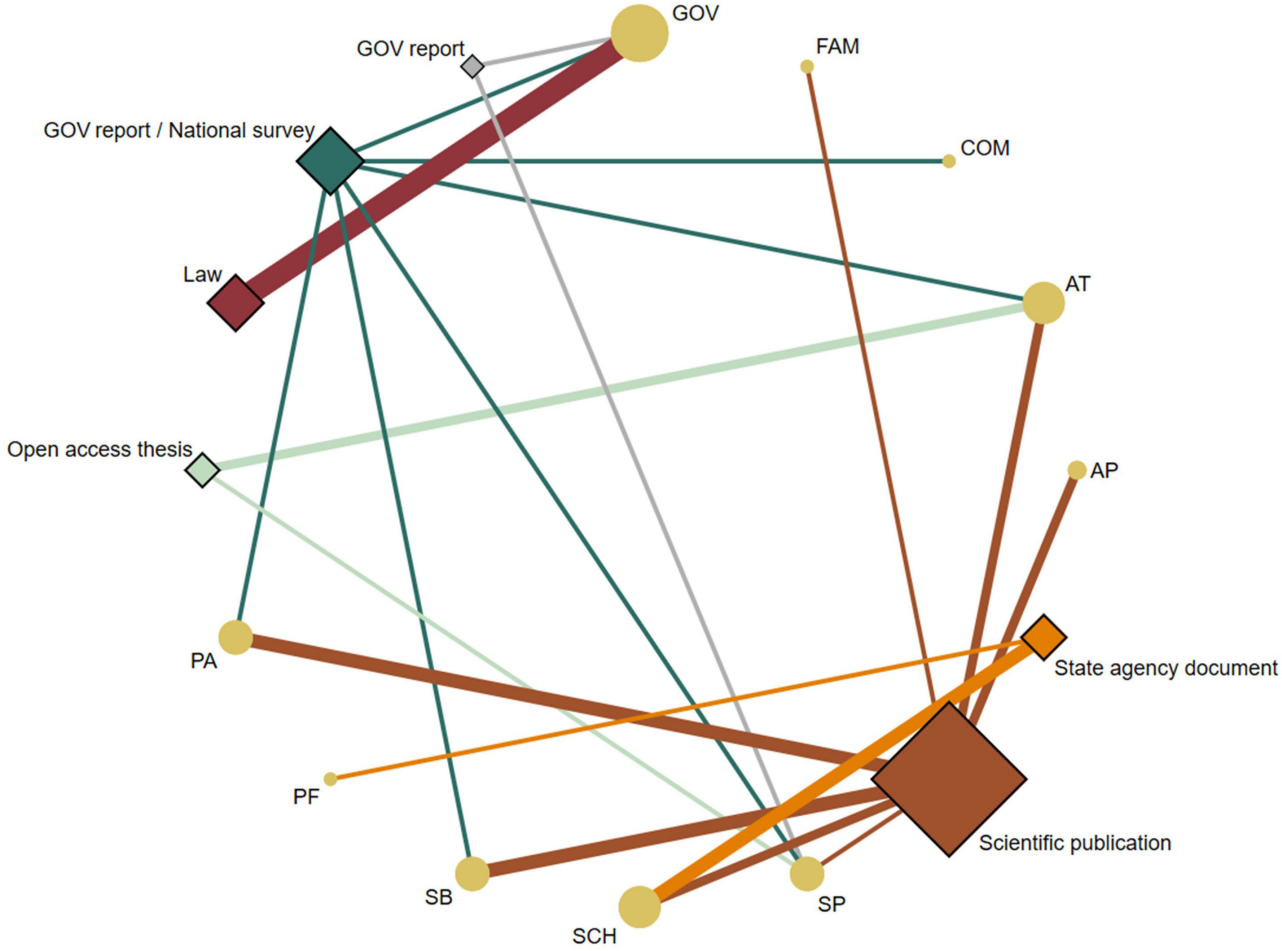
Network geometry plots of available comparisons between the PA-related indicators and the type of evidence. The size of the circular nodes (PA-related indicators) was relative to the number of papers analyzing these components. The size of the diamond nodes (type of evidence) was relative to the number of available data on PA-related indicators analyzing these components. The width of the solid line connecting the nodes was relative to the number of papers analyzing the PA-related indicators (circular nodes) according to the type of evidence (diamond nodes). AP, Active play; AT, Active transportation; COM, Community and built environment; FAM, Family and Peers; GOV, Government; PA, Physical activity; PF, Physical fitness; SB, Sedentary behavior; SCH, School environment; SP, Sport and physical activity participation.

Figure 3 depicts the PA-related indicators analyzed by the type of evidence. The most common PA-related indicator setting targeted in seven papers was GOV, including five laws (26–30) and two government reports (34, 36). Two peer-reviewed scientific papers (31, 32), two bachelor's thesis (42, 43), and one government report (10) focused on AT. Under SCH, two peer-reviewed scientific papers (25, 33) and three official documents of a State educational agency (38–40) were analyzed. Besides, four papers referred to PA or SB, including three peer-reviewed scientific papers (25, 31–33) and one government report (10). One peer-reviewed scientific paper (31), one bachelor's thesis (41), and two government reports (34, 36) focused on SP. Under AP, two peer-reviewed scientific papers (31, 33) were analyzed. Finally, under the COM, FAM, and PF indicators, a government report (35), a peer-reviewed scientific paper (25), and official documents of a State educational agency (37) were examined, respectively.

### Gender data

**Figure 4:**
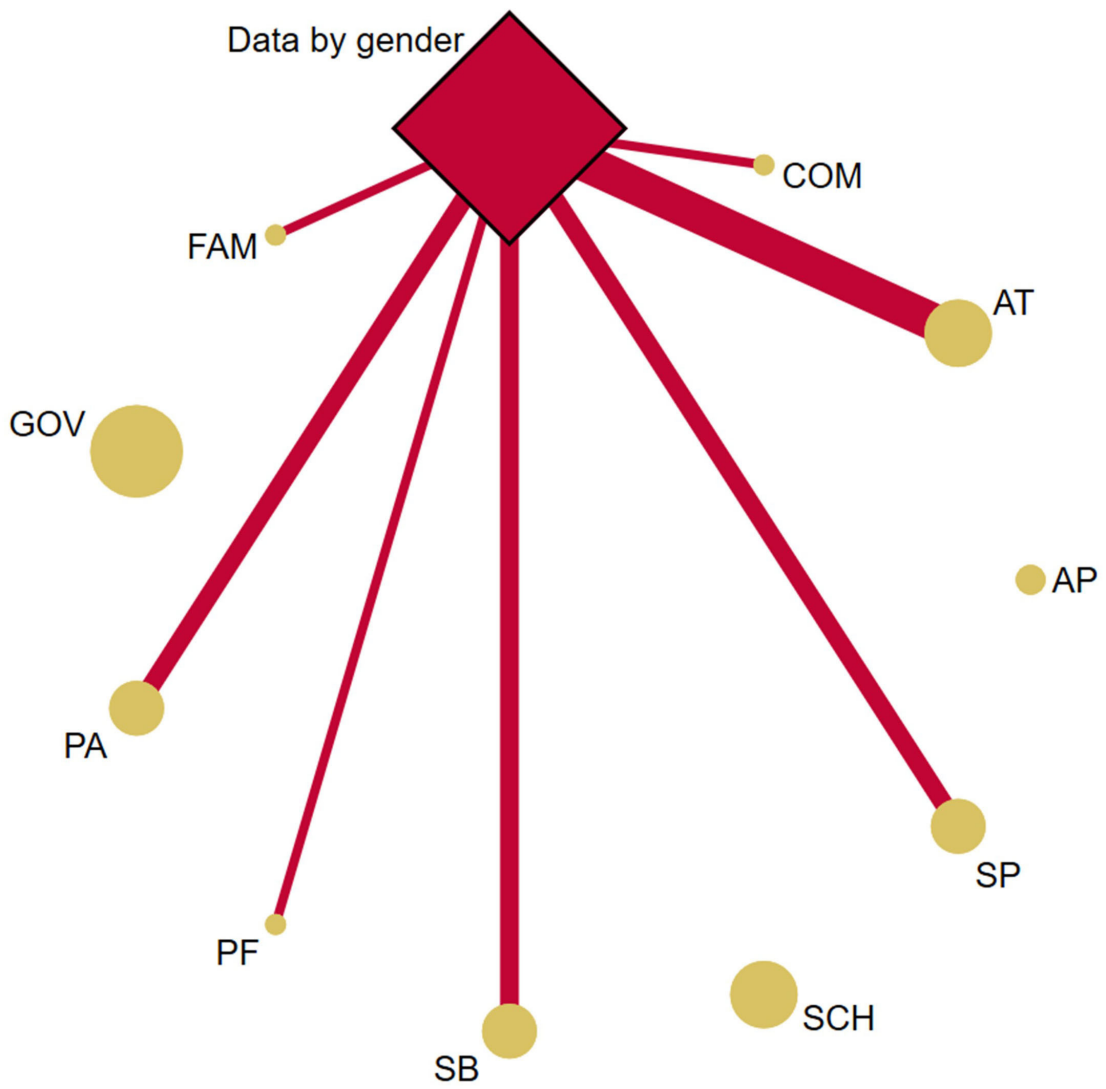
Network geometry plots of available comparisons between the PA-related indicators and gender data. The size of the circular nodes (PA-related indicators) was relative to the number of papers analyzing these components. The size of the diamond nodes (gender data) was relative to the number of available data on PA-related indicators analyzing these components. The width of the solid line connecting the nodes was relative to the number of papers analyzing the PA-related indicators (circular nodes) according to gender data (diamond nodes). AP, Active play; AT, Active transportation; COM, Community and built environment; FAM, Family and Peers; GOV, Government; PA, Physical activity; PF, Physical fitness; SB, Sedentary behavior; SCH, School environment; SP, Sport and physical activity participation.

Figure 4 displays the PA-related indicators analyzed by gender. Of the papers included (nine out of 20) that reported data by gender, the total number of children and adolescents involved was 150,012 (10, 25, 32, 34, 35, 37, 41–43). Overall, all these papers reported better results on PA-related indicator levels for boys compared to girls. However, favorable results for girls were established on AT (i.e., commuted actively to/from school), SB (i.e., screentime), and PF (i.e., flexibility) compared to boys in four papers (10, 32, 37, 42).

### Key findings from evidence

Compared to the 2018 Report Card (evidence searches from inception to 2018 included seven papers to report the indicator grades), the Uruguay 2022 Report Card (evidence searches between 2018 and 2021) was based on a larger number of papers (19 out of 20 included in this ScR).

**Figure 5:**
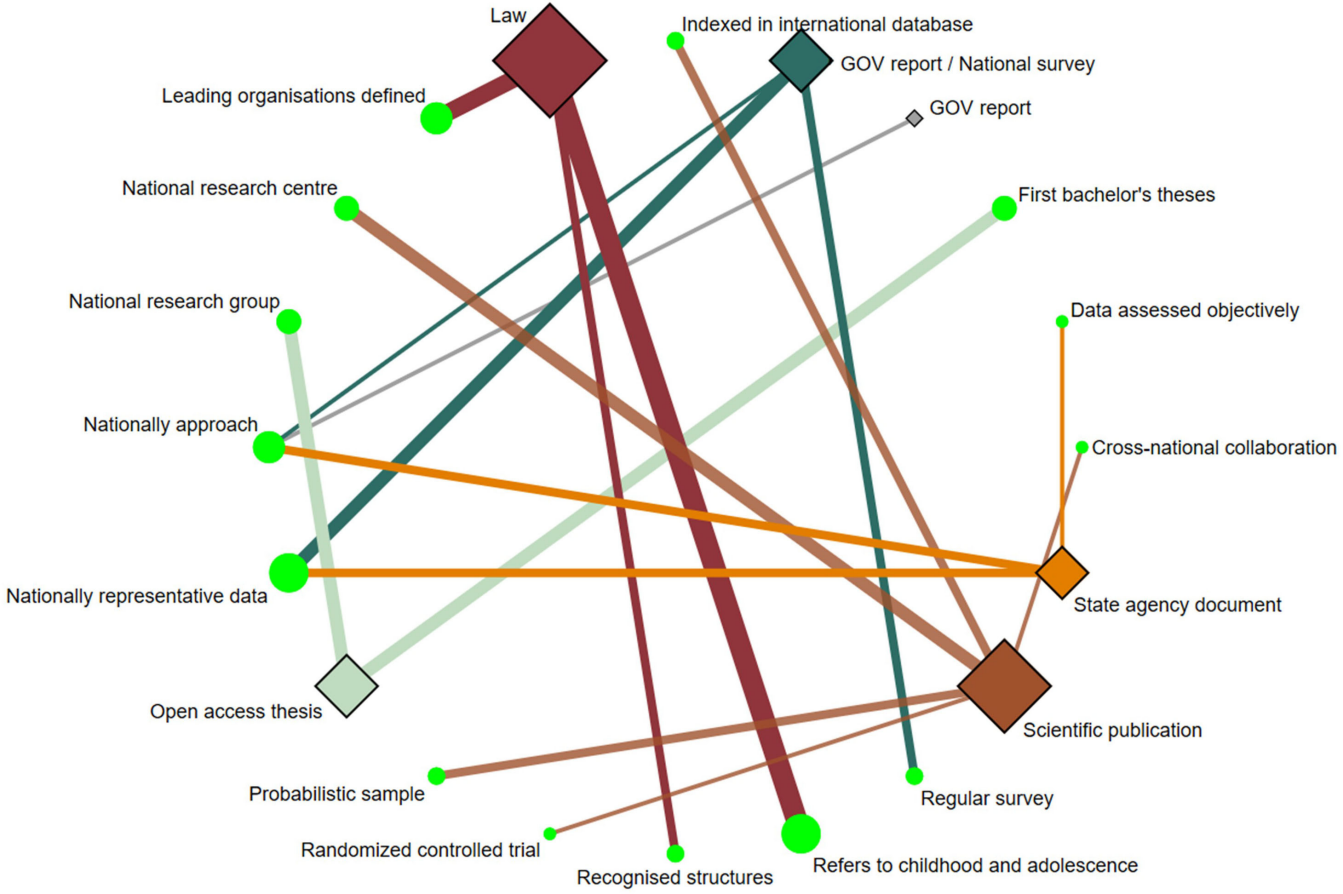
Network geometry plots of available comparisons between the type of evidence and their strengths. The size of the circular nodes (evidence strengths) was relative to the number of papers analyzing these components. The size of the diamond nodes (type of evidence) was relative to the number of available data on evidence strengths analyzing these components. The width of the solid line connecting the nodes was relative to the number of papers analyzing the evidence strengths (circular nodes) according to the type of evidence (diamond nodes). GOV, Government.

**Figure 6:**
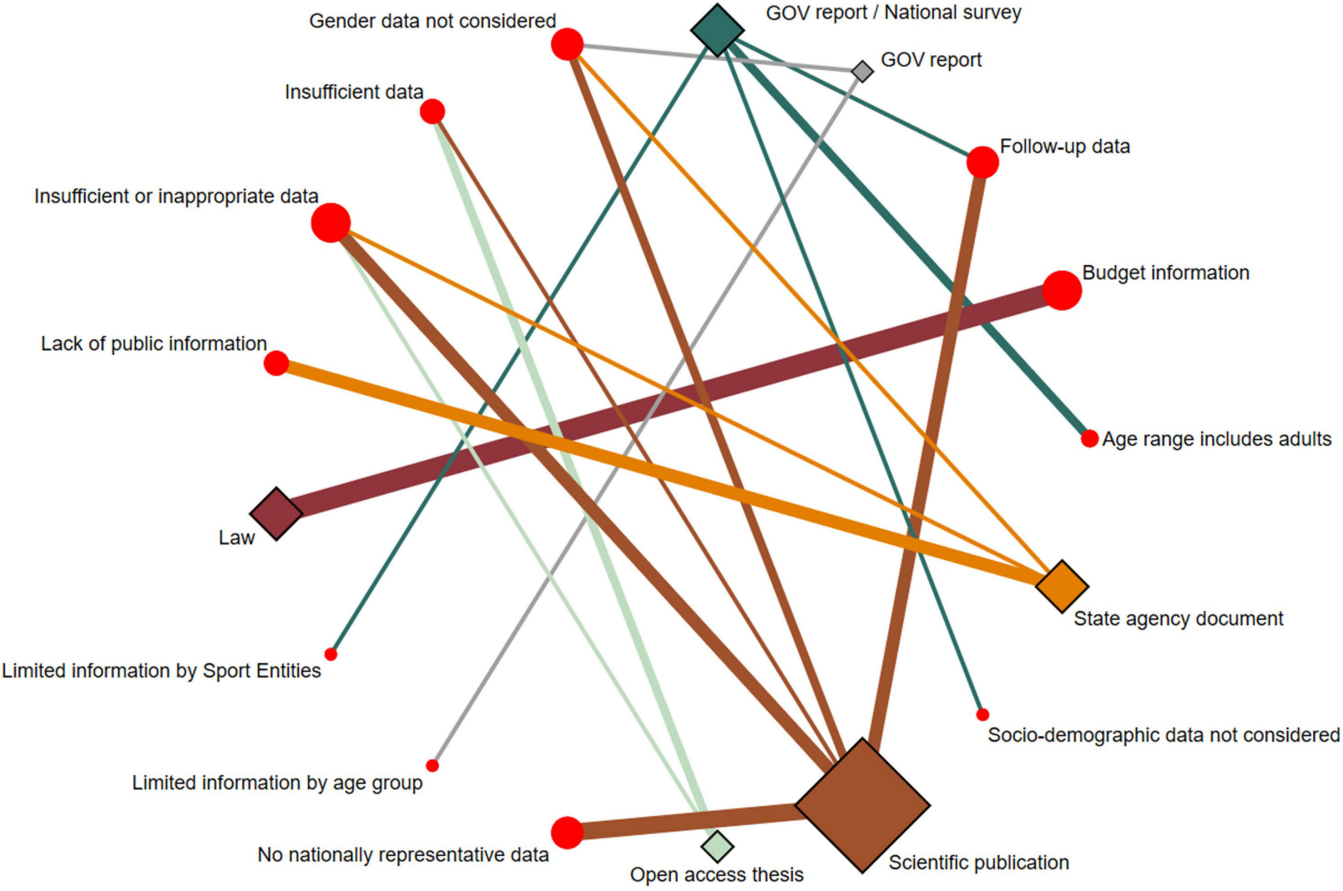
Network geometry plots of available comparisons between the type of evidence and their weaknesses. The size of the circular nodes (evidence weaknesses) was relative to the number of papers analyzing these components. The size of the diamond nodes (type of evidence) was relative to the number of available data on evidence weaknesses analyzing these components. The width of the solid line connecting the nodes was relative to the number of papers analyzing the evidence weaknesses (circular nodes) according to the type of evidence (diamond nodes). GOV, Government.

**Figure 7:**
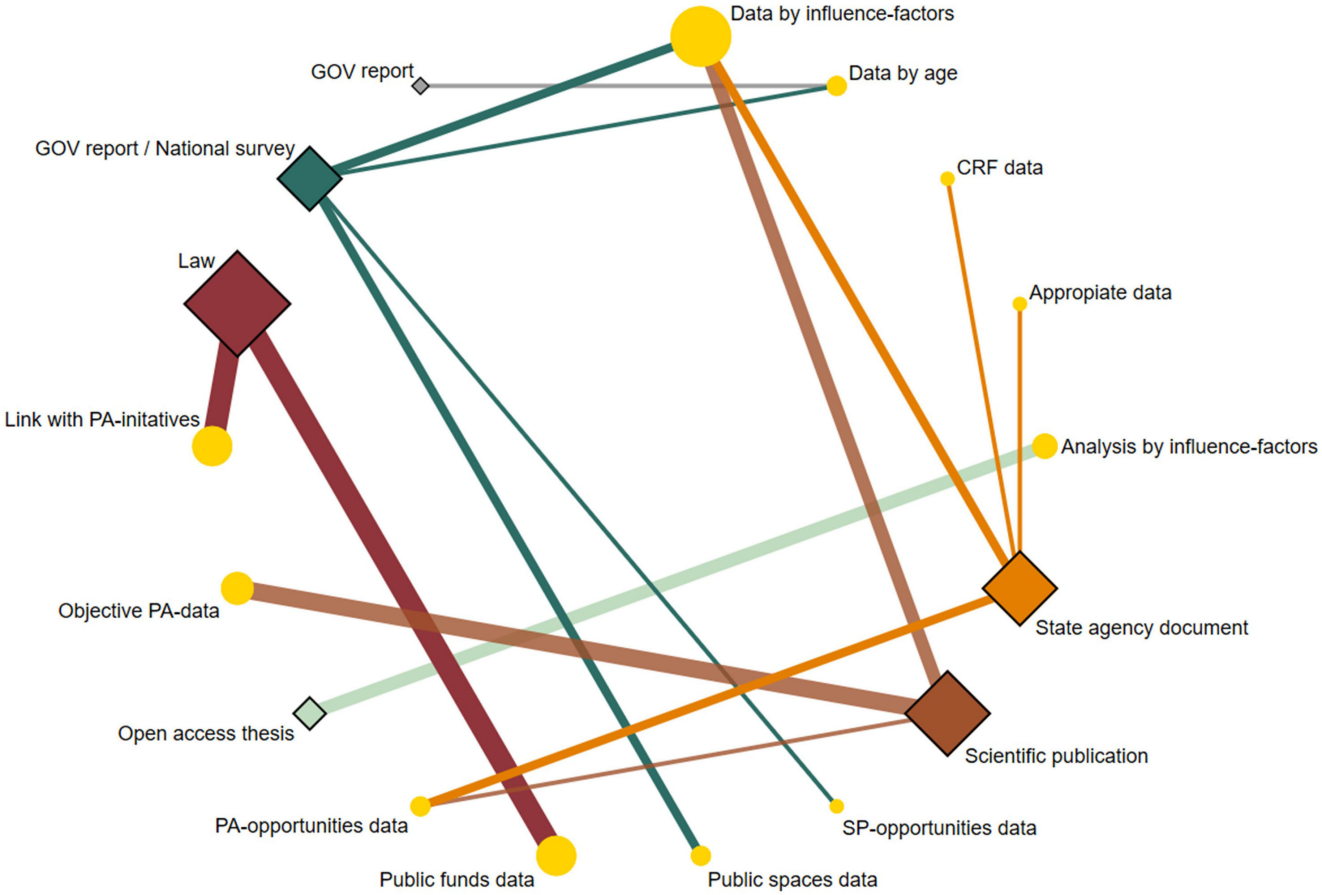
Network geometry plots of available comparisons between the type of evidence and their gaps. The size of the circular nodes (research gaps) was relative to the number of papers analyzing these components. The size of the diamond nodes (type of evidence) was relative to the number of available data on research gaps analyzing these components. The width of the solid line connecting the nodes was relative to the number of papers analyzing the research gaps (circular nodes) according to the type of evidence (diamond nodes). CRF, Cardiorespiratory fitness; GOV, Government; PA, Physical activity; SP, Sport and physical activity participation.

Supplementary Table S4 and Figures 5–7 detail the key findings from the included literature on PA-related indicators in Uruguayan children and adolescents.

### Evidence strengths/weaknesses/gaps

The strengths of the evidence from scientific publications were indexed in international databases (25, 33), randomized controlled trial approach (25), probabilistic study samples (25, 32, 33), leadership of national and interdisciplinary research centers (25, 33), and cross-national collaboration (25). Moreover, evidence weaknesses revealed the lack of nationally representative data (25, 31–33), insufficient or inappropriate information to report the PA-related indicator grades (25, 31–33), no follow-up data (31–33), and gender data not considered (31, 33). Finally, research gaps identified concern the influence of PA-related factors (e.g., gender and parents' education level) (25, 31–33), objective assessment of PA (25, 31–33), and information on PA opportunities at school in addition to physical education classes (25).

Regarding the papers included *via* other methods, the strengths of the evidence were nationally representative data (10, 34, 35, 37, 38), national approach framework (34, 36, 39, 40), periodic surveys (10, 34, 35), data assessed objectively (37), the first bachelor's theses based on the indicators of the GM initiative (41–43), leading organizations defined (26, 28–30) and recognized structures to create reports (26, 29), laws concerning PA in children and adolescents (26, 28–30), and national research groups (41–43). Moreover, evidence weaknesses revealed were the lack of public information (38–40), insufficient or inappropriate data to inform the PA-related indicator grades (37, 41–43), gender data not considered (36, 38), limited information (34, 36), no follow-up data (10, 34, 35), age range including adults (34, 35), no budget information (26–30), and socio-demographic data not considered (10). Finally, research gaps identified concern the influence of PA-related factors (e.g., country's regions and socioeconomic status) (10, 35, 38–43), information on PA opportunities at school in addition to physical education classes (39, 40), data on SP opportunities at the community in addition to federated sports (34), findings on SP policies and COM according to specific youth ages (35, 36), appropriate data on PF (37), systematized information on public spaces and infrastructure according to PA-related indicators (35), accessible information on public funds (26–30, 35), and links between laws and PA initiatives (26–30).

## Discussion

This is the first ScR summarizing the evidence status of PA-related indicators in Uruguayan children and adolescents. Strengths, weaknesses, and knowledge gaps were identified and synthesized based on the literature. Overall, findings from our review indicate that scientific evidence and the research productivity in PA-related indicators in Uruguay are scarce and with different shortcomings; however, there have been some advances in this topic, such as the increase in research evidence compared to Uruguay's 2018 Report Card, which is worth highlighting. Furthermore, synthesizing both weaknesses and knowledge gaps allowed for the identification of future research perspectives.

Our review detailed the emerging field of PA-related indicators research in Uruguay over the last 4 years, including scientific publications, open access thesis, government reports, national surveys, and official documents of State-agencies. Overall, a greater number of papers (*n* = 19) with updated data on the PA-related indicators were found compared to Uruguay's 2018 Report Card (*n* = 8) (44). Although some of these data were inappropriate (e.g., small study sample and lack of public information) for reporting indicator grades, we found evidence of some advances in this research field in Uruguay that are important to emphasize. This agrees with the Latin American context, where PA research is still an emerging field but with some important progress in research capacity in recent years (17).

Considering the scientific papers included, our review revealed the first scientific papers indexed in international databases that analyze PA-related indicators in Uruguayan childhood or adolescence (25, 33). This is an important finding of the last 4 years compared to Uruguay's 2018 Report Card, where only scientific papers published in national journals were included (15). In fact, a systematic review showed that until 2015, there were no scientific publications or cross-national collaborations (16) and in 2017, the Global Observatory of Physical Activity confirmed the lack of research initiatives (45). Results of our ScR suggest that a small step forward in scientific knowledge on PA-related indicators has been made recently. Research productivity through high-impact peer-reviewed scientific publications is a relevant indicator of research capacity (17).

Moreover, two national interdisciplinary research centers (25, 33) and one cross-national collaboration (25) represent major strength opportunities for research development. These initiatives are crucial for capacity building, promoting a research environment conducive to multidisciplinary collaboration, and making better use of financial and human resources (16). Although incipiently, this allows the development of different research capacity components (17), such as the consolidation of research teams (25, 33, 46), local training programs (47), networking opportunities (47, 48), and cross-national collaborations (46, 49–51). These points are especially important for building a sustainable research agenda in Uruguay, whereas research capacity and resources are limited (18), and the investigation of PA-related indicators is a nascent field (17).

Considering papers other than scientific publications, our results indicate a major strength with three nationally representative surveys that were conducted periodically (10, 34, 35). Effective public health programs and policies require contextually relevant evidence (52). For this purpose, nationally representative data are crucial and should be a priority for designing and implementing local policies (53). The major challenge is to link an integral context-based approach to research with the translation of findings into the implementation of multi-component and multi-sectoral policies for tackling the physical inactivity pandemic (53). Additionally, the first Uruguayan bachelor's theses (41–43) based on the indicators of the GM initiative represent a huge step forward, which will be better reflected in the coming years as research work is further developed and eventually published. Finally, regarding the consolidation of national research, the theses research was performed with the support of a national research group with several cross-national collaborations and scientific publications (15, 46, 54, 55).

Regarding the PA-related indicators, our review displayed a nationally approached framework for SP (federated sports) and SCH (physical education classes) standards. Uruguay's National Sport Plan 2015–2020 (36) and the National Observatory of Sport (34) provided updated data on policies, programs, projects, funding, rationale, actions, and epidemiological characterization of SP. Meanwhile, the National Public Education Administration (37–40) reported updated data on SCH, where physical education is mandatory in formal education. Schools are ideally placed to promote PA strategies and to provide an increment in PA levels (56). Particularly in Uruguay, where primary and secondary education showed universal (around 99% of children aged 6–11) and high (around 98.2% of adolescents aged 12–14 years and 92.3% of adolescents aged 15–17 years) coverage, respectively, in 2020 (57). Additionally, different laws link both SP and SCH indicators with governmental functioning and rationale (26, 28–30). Therefore, these nationally approached frameworks are strengths for PA opportunities in Uruguayan youths. Moreover, they represent an important research focus that should be further developed not only in physical education classes but also in federated sports participation.

Beyond these advances, our ScR revealed that papers examining the PA-related indicators in Uruguayan children and adolescents remain lacking and high-quality research production is a major challenge. Indeed, nearly half of the indicators (i.e., AT, PA, and SB) in Uruguay's 2022 Report Card (19) were graded based on the WHO Global School-based Student Health Survey (10) because of the lack of other nationally representative studies. This is an indication that Uruguay has an issue with the lack of capacity required for high-quality research and/or is still looking to understand the extent of the problem and recognize the importance of PA at policy and academia levels (16).

The few scientific productions, the small number of cross-national collaborations and research centers, the limited data (availability, access, and quality), and the near absence of papers published on some PA-related indicators (i.e., AP, COM, FAM, and PF) display a void in the research capacity concerning this topic in Uruguay. Some weaknesses reported in the Latin American context (16, 58) could also influence national research capacity: few graduate-level training programs on PA and health, a limited number of researchers specifically trained to assess PA as a health issue, deficient English language proficiency, or low resources to conduct research projects and publish collected data. Based on the results of this ScR and the stages of the behavioral epidemiology framework (59), the level of maturity of PA research in Uruguay is emerging and urgently demands for further growth.

Results of our ScR suggest that there are many areas to contemplate in future PA research. Specifically, social inequalities remain among the biggest challenges for global PA promotion (53) and should be a priority at the national level. Socioeconomical status and gender are substantial inequalities across the PA-related indicators, with the poorest and girls being the least active during leisure time (60–62). Resolving socioeconomic and gender-based inequalities could help improve PA levels in children and adolescents, and conversely, PA promotion strategies can reduce social inequalities (53). High-quality research can lead to significant evidence for initiatives that work at scale (63), playing an important role in achieving a reduction in inequality (53). The promotion of equity can happen at schools, transport systems, urban environments and designs, comprehensive community action, and sports promotion (53). However, to support these strategies that affect equitable distribution, research capacity needs to be improved. This would provide credible and appropriate evidence on adherence to PA guidelines by social inequality indicators. Globally (64) and specifically in Uruguay, this remains an important research gap that should be addressed in future papers for the better development of PA strategies.

### Summary of key recommendations

The authors recommend that national efforts on PA-related indicators research should be made to address two interrelated priority paths.

On the one hand, related to weaknesses in the published literature, they should include scientific publications, specifically with nationally representative data and longitudinal analysis; cross-national and cross-sectoral collaborations in research projects; creation and consolidation of national and transdisciplinary research centers, mostly in cities that have been underrepresented compared to the Uruguayan capital; and national surveys with follow-up data.

On the other hand, related to knowledge gaps in the PA-related indicators, they should include: appropriate data, particularly on AP, COM, FAM, PF, and SP; information by gender, particularly on AP, SCH, and GOV; evidence by influence factors (e.g., country's regions, socioeconomic status); accessible public information, principally on GOV (e.g., information on policies, programmes and funding for the implementation of PA promotion strategies) and SCH (e.g., active school policies and description of their approach); data by specific children ages, mainly on AT, PA, and SB; knowledge about a whole-school approach that includes components such as modified school policies to engage students with low PA levels or parental engagement; findings on SP in community environments; evidence on public spaces and infrastructure; and device-based PA data.

More specifically, the authors recommend that future research initiatives on PA-related indicators should incorporate the methodological GM framework and report data on this basis. This would help improve epidemiological characterization and, therefore, guide policy development to increase PA levels. We also encourage future research projects to have a comprehensive approach that assesses relevant PA-related factors of influence, such as gender or socioeconomic status, for a better reflection of the local context. This information will also be key to developing efficient policies encouraging PA. Finally, the authors call for a synergistic approach to future research proposals that will allow further progress in an emerging and challenging scientific field in Uruguay. Specifically, research strategies emphasizing multicomponent (e.g., daily behaviors, contexts, and sources of influence), cross-sectorial (e.g., transdisciplinary investigations and academic-government approaches), and cross-national (e.g., training programs, material resources collaborations) initiatives will be key to increasing the level of maturity of PA research on children and adolescents and to guide effective policy actions based on high-quality evidence.

### Strengths and limitations

This study is the first to provide an ScR on PA-related indicators in children and adolescents from Uruguay. The methodological framework used could be useful for countries with a lack of research on this topic and for new countries in future editions of the GM initiative. Regarding the Uruguayan context, the above-mentioned strengths in PA-related indicators research and evidence are crucial advances to improve research capacity and guide effective policy actions.

Our study has limitations that should be acknowledged. Because we conducted an ScR, it is not necessary to rate the quality of the data or conduct a critical appraisal of the evidence included. Furthermore, although we used a broad search strategy, it is possible that some papers were missed. Additionally, no qualitative papers or papers reporting data on specific groups of interest (e.g., functional disabilities) were included, which could exclude relevant data when mapping Uruguay's PA research capacity. We intended to provide an overall picture of the state of the PA-related indicators research in Uruguay based on the GM initiative.

## Conclusion

This is the first ScR generating a comprehensive view of the physical activity evidence in children and adolescents from Uruguay. Given the lack of previous papers at the national level, our findings provide the best available evidence for identifying and overcoming the challenges of physical activity-related indicators research. Understanding strengths, weaknesses, and research gaps are essential to improve research capacity in this health behavior field. Although papers examining physical activity-related indicators in Uruguayan children and adolescents remain lacking, we displayed some advances in research production. From the public health perspective, the double strength is clear: by improving research capacity, high-quality evidence becomes available to guide effective policy action in Uruguay. Knowing the epidemiological reality should be the main objective in future research to identify evidence-based challenges and priorities for action on physical activity-related indicators. The methodological framework applied could be useful for countries of the Global Matrix initiative.

## Author contributions

BB-P and JB-S: conceptualization, methodology, formal analysis, investigation, data curation, writing—original draft, writing—review and editing, and visualization. JB-S: supervision. CAC, EP-T, SF-G, and VD-G: data curation and writing—review and editing. All authors contributed to the article and approved the submitted version.

## Funding

Processing Charges have been covered by the PDU EFISAL—Universidad de la República (Exp. n° 003051-000603-16).

## Conflict of interest

The authors declare that the research was conducted in the absence of any commercial or financial relationships that could be construed as a potential conflict of interest.

## Publisher's note

All claims expressed in this article are solely those of the authors and do not necessarily represent those of their affiliated organizations, or those of the publisher, the editors and the reviewers. Any product that may be evaluated in this article, or claim that may be made by its manufacturer, is not guaranteed or endorsed by the publisher.
