# Supplementary material for: Physical activity-related indicators in children and adolescents in Uruguay: A scoping review based on the Global Matrix initiative
Source: Front Public Health. 2022 Sep 7;10:954621. doi: 10.3389/fpubh.2022.954621 (PMC9490234; doi:10.3389/fpubh.2022.954621)
Supplement: Supplementary file 1 [file Table_1.docx]

Supplementary Material

# Supplementary Tables

**Table S1.** PRISMA checklist for scoping reviews.

| **SECTION** | **ITEM** | **PRISMA CHECKLIST ITEM** | **REPORTED ON PAGE #** |
| --- | --- | --- | --- |
| **TITLE** | | | |
| Title | 1 | Identify the report as a scoping review. | 1 |
| **ABSTRACT** | | | |
| Structured summary | 2 | Provide a structured summary that includes (as applicable): background, objectives, eligibility criteria, sources of evidence, charting methods, results, and conclusions that relate to the review questions and objectives. | 1-2 |
| **INTRODUCTION** | | | |
| Rationale | 3 | Describe the rationale for the review in the context of what is already known. Explain why the review questions/objectives lend themselves to a scoping review approach. | 3 |
| Objectives | 4 | Provide an explicit statement of the questions and objectives being addressed with reference to their key elements (e.g., population or participants, concepts, and context) or other relevant key elements used to conceptualize the review questions and/or objectives. | 2 |
| **METHODS** | | | |
| Protocol and registration | 5 | Indicate whether a review protocol exists; state if and where it can be accessed (e.g., a Web address); and if available, provide registration information, including the registration number. | 3 |
| Eligibility criteria | 6 | Specify characteristics of the sources of evidence used as eligibility criteria (e.g., years considered, language, and publication status), and provide a rationale. | 3 |
| Information sources | 7 | Describe all information sources in the search (e.g., databases with dates of coverage and contact with authors to identify additional sources), as well as the date the most recent search was executed. | 3-4 |
| Search | 8 | Present the full electronic search strategy for at least 1 database, including any limits used, such that it could be repeated. | 3-4 |
| Selection of sources of evidence | 9 | State the process for selecting sources of evidence (i.e., screening and eligibility) included in the scoping review. | 3-4 |
| Data charting process | 10 | Describe the methods of charting data from the included sources of evidence (e.g., calibrated forms or forms that have been tested by the team before their use, and whether data charting was done independently or in duplicate) and any processes for obtaining and confirming data from investigators. | 4 |
| Data items | 11 | List and define all variables for which data were sought and any assumptions and simplifications made. | 4 |
| Critical appraisal of individual sources of evidence | 12 | If done, provide a rationale for conducting a critical appraisal of included sources of evidence; describe the methods used and how this information was used in any data synthesis (if appropriate). | - |
| Synthesis of results | 13 | Describe the methods of handling and summarizing the data that were charted. | 4 |
| **RESULTS** | | | |
| Selection of sources of evidence | 14 | Give numbers of sources of evidence screened, assessed for eligibility, and included in the review, with reasons for exclusions at each stage, ideally using a flow diagram. | 5 |
| Characteristics of sources of evidence | 15 | For each source of evidence, present characteristics for which data were charted and provide the citations. | 5 |
| Critical appraisal within sources of evidence | 16 | If done, present data on critical appraisal of included sources of evidence (see item 12). | - |
| Results of individual sources of evidence | 17 | For each included source of evidence, present the relevant data that were charted that relate to the review questions and objectives. | 5-6 |
| Synthesis of results | 18 | Summarize and/or present the charting results as they relate to the review questions and objectives. | 6-7 |
| **DISCUSSION** | | | |
| Summary of evidence | 19 | Summarize the main results (including an overview of concepts, themes, and types of evidence available), link to the review questions and objectives, and consider the relevance to key groups. | 7-10 |
| Limitations | 20 | Discuss the limitations of the scoping review process. | 10 |
| Conclusions | 21 | Provide a general interpretation of the results with respect to the review questions and objectives, as well as potential implications and/or next steps. | 10 |

**Table S2.** Search strategy detailed information.

| **Source** | **Search terms** |
| --- | --- |
| PubMed | (“physical activ*” OR “motor activ*” OR movement OR “physical education” OR “physical inactiv*” OR sitting OR sedentary* OR “active travel” OR “active transportat*” OR “active commut*” OR “outdoor play” OR “active play” OR outdoor* OR play* OR fitness OR “physical fitness” OR “physical performance” OR “muscle strength” OR cardiorespiratory OR “aerobic capacity” OR sport* OR policy* OR legislation OR strategy OR “national framework” OR “built environment*” OR walkabil* OR bikeabil* OR neighborhood* OR “urban parks” OR facilitator OR support OR community OR municipality OR school* OR playground OR classroom* OR recess OR family OR parent* OR peer* OR “healthy lifestyle” OR wellness) AND (child* OR adolescen* OR youth* OR minor OR pediatric* OR teen* OR “school age”) AND (Uruguay*) |
| Web of Science | (“physical activ*” OR “motor activ*” OR movement OR “physical education” OR “physical inactiv*” OR sitting OR sedentary* OR “active travel” OR “active transportat*” OR “active commut*” OR “outdoor play” OR “active play” OR outdoor* OR play* OR fitness OR “physical fitness” OR “physical performance” OR “muscle strength” OR cardiorespiratory OR “aerobic capacity” OR sport* OR policy* OR legislation OR strategy OR “national framework” OR “built environment*” OR walkabil* OR bikeabil* OR neighborhood* OR “urban parks” OR facilitator OR support OR community OR municipality OR school* OR playground OR classroom* OR recess OR family OR parent* OR peer* OR “healthy lifestyle” OR wellness) AND (child* OR adolescen* OR youth* OR minor OR pediatric* OR teen* OR “school age”) AND (Uruguay*) |
| LILACS | (“physical activ*” OR “motor activ*” OR movement OR “physical education” OR “physical inactiv*” OR sitting OR sedentary* OR “active travel” OR “active transportat*” OR “active commut*” OR “outdoor play” OR “active play” OR outdoor* OR play* OR fitness OR “physical fitness” OR “physical performance” OR “muscle strength” OR cardiorespiratory OR “aerobic capacity” OR sport* OR policy* OR legislation OR strategy OR “national framework” OR “built environment*” OR walkabil* OR bikeabil* OR neighborhood* OR “urban parks” OR facilitator OR support OR community OR municipality OR school* OR playground OR classroom* OR recess OR family OR parent* OR peer* OR “healthy lifestyle” OR wellness) AND (child* OR adolescen* OR youth* OR minor OR pediatric* OR teen* OR “school age”) AND (Uruguay*) |
| LILACS | ("actividad física" OR "actividad motora" OR movimiento OR "educación física" OR "inactividad física" OR sedentario OR "transporte activo" OR "desplazamiento activo" OR "juego al aire libre" OR "juego activo" OR “aire libre” OR juego OR fitness OR "condición física" OR "rendimiento físico" OR "fuerza muscular" OR cardiorespiratorio OR "capacidad aeróbica" OR deporte OR política OR legislación OR estrategia OR "marco nacional" OR "entorno construido" OR vecindario OR "parque urbano" OR facilitador OR soporte OR comunidad OR municipalidad OR escuela OR "parque infantil" OR aula OR receso OR familia OR padres OR pares OR "estilo de vida saludable" OR salud) AND (niño OR adolescente OR jóvenes OR menor OR pediátrico OR "edad escolar") AND (Uruguay*) |
| Scielo | (“physical activ*” OR “motor activ*” OR movement OR “physical education” OR “physical inactiv*” OR sitting OR sedentary* OR “active travel” OR “active transportat*” OR “active commut*” OR “outdoor play” OR “active play” OR outdoor* OR play* OR fitness OR “physical fitness” OR “physical performance” OR “muscle strength” OR cardiorespiratory OR “aerobic capacity” OR sport* OR policy* OR legislation OR strategy OR “national framework” OR “built environment*” OR walkabil* OR bikeabil* OR neighborhood* OR “urban parks” OR facilitator OR support OR community OR municipality OR school* OR playground OR classroom* OR recess OR family OR parent* OR peer* OR “healthy lifestyle” OR wellness) AND (child* OR adolescen* OR youth* OR minor OR pediatric* OR teen* OR “school age”) AND (Uruguay*) |
| Scielo | ("actividad física" OR "actividad motora" OR movimiento OR "educación física" OR "inactividad física" OR sedentario OR "transporte activo" OR "desplazamiento activo" OR "juego al aire libre" OR "juego activo" OR “aire libre” OR juego OR fitness OR "condición física" OR "rendimiento físico" OR "fuerza muscular" OR cardiorespiratorio OR "capacidad aeróbica" OR deporte OR política OR legislación OR estrategia OR "marco nacional" OR "entorno construido" OR vecindario OR "parque urbano" OR facilitador OR soporte OR comunidad OR municipalidad OR escuela OR "parque infantil" OR aula OR receso OR familia OR padres OR pares OR "estilo de vida saludable" OR salud) AND (niño OR adolescente OR jóvenes OR menor OR pediátrico OR "edad escolar") AND (Uruguay*) |
| Latindex | ("actividad física" OR sedentario OR desplazamiento OR juego OR fitness OR deporte OR política OR comunidad OR escuela OR padres) AND (niño OR adolescente OR infancia) AND (Uruguay*) |
| Specific searches ^a^ | “Lecturas: Educación Física y Deportes”, “Revista Argentina de Medicina”, “Sociedad Argentina de Pediatría”, “Revista Brasileña de Ciencias del Deporte”, “Revista Brasileña de Actividad Física y Salud”, “Archivos de Pediatría del Uruguay”, “Revista Universitaria de la Educación Física y el Deporte”, “Revista Médica del Uruguay”, “Revista Uruguaya de Enfermería”, “Anales de la Facultad de Medicina”, “Revista Uruguaya de Cardiología”, “Timbó” database |

^a^ The searches were performed in national (database) and regional relevant journals between 2018 (date of the first review on PA-related indicators evidence through Uruguay’s 2018 Report Card) and 2021.

**Table S3.** Characteristics of included studies.

|  | **Studies characteristics** |  | **Participant’s characteristics** | | | **PA analysis** |
| --- | --- | --- | --- | --- | --- | --- |
| **Ref.** | **Author (year)** | **Study design** | **Sample (*n*)** | **Age range (*y*)** | **Girls (*%*)** | **Indicators** |
|  | *Studies via databases* |  |  |  |  |  |
| (32) | Bove et al. (2020) | Peer-reviewed scientific paper | 771 | 5–6 | 48.8 | AT, PA, SB |
| (33) | Gómez et al. (2021) | Peer-reviewed scientific paper | 816 | 5–6 | NR | AP, PA, SB, SCH |
| (31) | Machado et al. (2018) | Peer-reviewed scientific paper | 318 | 9–12 | 52.6 | AP, AT, SB, SP |
| (25) | Springer et al. (2019) | Peer-reviewed scientific paper | 654 | 11–15 | 50.6 | FAM, PA, SCH |
|  | *Studies via other methods* |  |  |  |  |  |
| (42) | Da Silva et al. (2021) | Open access bachelor's thesis | 55 | 6–12 | 54.5 | AT, COM |
| (43) | Fernández et al. (2019) | Open access bachelor's thesis | 70 | 6–10 | 47.2 | AT, COM |
| (38) | National Public Education Administration (2020) | Official document from State-agency | 76510 | NR ^a^ | NR | SCH |
| (39) | National Public Education Administration (2014) | Official document from State-agency | NA | NA ^a^ | NA | SCH |
| (40) | National Public Education Administration (2020) | Official document from State-agency | NA | NA ^b^ | NA | SCH |
| (36) | National Secretary of Sport (2020) | Government report | NA | NA | NA | GOV, SP |
| (34) | National Secretary of Sport (2019) | Government report / National survey | 136483 ^c^ | 6–19 | 13.6 | GOV, SP |
| (37) | National Secretary of Sport / National Public Education Administration (2018) | Official document from two State-agencies | 6755 | 10–14 | 49.9 | PF |
| (35) | National Youth Institute (2020) | Government report / National survey | 6534  2007 | 12-35  12–17 | 53.1 ^d^ | COM |
| (41) | Prates et al. (2021) | Open access bachelor's thesis | 55 | 6–12 | 54.5 | SP |
| (30) | Uruguayan Parliament (2007) | Law (n° 18213) | NA | NA ^a^ | NA | GOV |
| (26) | Uruguayan Parliament (2008) | Law (n° 18437) | NA | NA | NA | GOV |
| (29) | Uruguayan Parliament (2011) | Law (n° 18833) | NA | NA | NA | GOV |
| (28) | Uruguayan Parliament (2019) | Law (n° 19828) | NA | NA | NA | GOV |
| (27) | Uruguayan Parliament (2020) | Law (n° 19889) | NA | NA | NA | GOV |
| (10) | World Health Organization (2019) | Government report / National survey | 3162 | 13–17 | NR | AT, PA, SB |

^a^ Students enrolled on Initial and Primary Education (typical age range: 4–12 years). ^b^ Students enrolled on Secondary Education (typical age range: 13–17 years). ^c^ Partial data according to the Sports Entities (37 of 63) that reported information. ^d^ Data according to the total national survey sample (*n*=6534).

Abbreviations: AP, Active play; AT, Active transportation; COM, Community and built environment; FAM, Family and Peers; GOV, Government; NA, not applicable; NR, not reported; PA, overall physical activity; PF, Physical fitness; SB, Sedentary behaviour; SCH, School environment; SP, organised sport and physical activity participation.

**Table S4.** Synthesis of findings from included studies reporting physical activity-related indicators for Uruguayan children and adolescents.

| Study | PA-related indicators findings | Gender analysis | Key findings from evidence: strengths / weaknesses / gaps |
| --- | --- | --- | --- |
| *Studies via databases* |  |  |  |
| Bove et al. (2020) | *AT*- 79.3% of children aged 5–6 years commuted active (walk) to school at least one day a week.  *AT*- 22.8% of children aged 5–6 years commuted active (bike) to school at least one day a week.  *PA*- 41.7% of children aged 5–6 years meet the PA international guidelines ^a^.  *SB*- 39.6% of children aged 5–6 years reported less than 2 hours per day of screentime. | 79.3% for boys  80.2% for girls  23.8% for boys  21.7% for girls  23.8% for boys  21.7% for girls  39.5% for boys  39.7% for girls | Cross-sectional study published in a national journal and conducted with a probabilistic sample of 771 children of 24 public Primary schools from Montevideo city in 2016–2017. National and interdisciplinary centre for health research (CUiiDARTE) reporting updated data on the PA-related indicators based on the analysis of overweight/obesity and blood pressure as the main topics. / Participants were only from one city. The available data were insufficient to inform the indicator grades. / Influence of PA-related factors (e.g., obesity, parents' education). PA-related indicators evaluated objectively. |
| Gómez et al. (2021) | *AP*- children aged 5–6 years with a mean of 238 minutes per week of AP.  *PA*- children aged 5–6 years with a mean of 448 minutes per week of PA.  *SB*- children aged 5–6 years with a mean of 255 minutes per day of SB.  *SCH* - children aged 5–6 years with a mean of 40 minutes per week of PA at school. | N/A | Cross-sectional study published in an international journal and performed with a probabilistic sample of 816 children attending public Primary schools from Montevideo city in 2016. National and interdisciplinary centre for health research (CUiiDARTE) focused on cardiovascular system and reporting updated data on the PA-related indicators. / Participants were only from one city. The available data were insufficient or inappropriate to inform the indicator grades. / Influence of PA-related factors (e.g., gender, obesity). PA-related indicators evaluated objectively. |
| Machado et al. (2018) | *AP*- 81,9% of children aged 8–13 played outdoors more than 30 minutes per day.  *AT*- 61.0% of children aged 8–13 years commuted active (walk) more than 5 blocks per day.  *SB*- 9.3% of children aged 8–13 years reported less than 2 hours per day of screentime.  *SP*- 37.4% of children aged 8–13 years practiced more than 4 hours per week of programmed PA. | N/A | Cross-sectional study published in a national journal and performed with 318 children (179 with healthy body weight) of 12 public and private Primary schools from Montevideo and Rivera cities in 2015–2016. Medical researchers reporting updated data on the PA-related indicators based on the analysis of overweight/obesity as the main topic. / Convenience sample that included only participants from two cities. The available data were insufficient or inappropriate to inform the indicator grades. / Influence of PA-related factors (e.g., diet, obesity). PA-related indicators evaluated objectively. |
| Springer et al. (2019) | *FAM*- 68.2% of youth aged 11–15 years with friends who support them to be physically active.  *PA*- youth aged 11–15 years meet the PA international guidelines ^a^ during 3.4 days in the past week.  *SCH* - youth aged 11–15 years participated in PA at school (PE lessons or other PA occasions) during 6.2 number of times in the past week. | 74.9% for boys  61.8% for girls  N/A  N/A | Randomized controlled trial published in an international journal and performed with 654 youth of 16 public and private Secondary schools from Montevideo and Canelones cities in 2016–2017. National and interdisciplinary centre for health research (CIET) focused on tobacco prevention and reporting updated data on the PA-related indicators. Collaboration of an international health research centre. / Participants were only from two cities. The available data were insufficient or inappropriate to inform the indicator grades. / Evidence on PA opportunities at school in addition to PE classes with objective measures. |
| *Studies via other methods* |  |  |  |
| Da Silva et al. (2021) | *AT*- 47.3% of children aged 6–12 years commuted active (walk or bike) in the past week.  *AT*- 13.0% of children aged 6–12 years commuted active to school in the past week.  *AT*- 13.0% of children aged 6–12 years commuted active from school in the past week. | 48.0% for boys  46.7% for girls  24.0% for boys  3.4% for girls  12.0% for boys  13.8% for girls | First bachelor's theses on PA-related indicators of the GM initiative. Cross-sectional study indexed in the official repository of the University of the Republic and performed with 55 children of a private Primary school from Rivera city in 2020. National research group (GIARH) focused on PA and health. / Participants were only from one Uruguayan city and the study sample was small. The available data were insufficient to report a grade. / Data according to different AT-related facilitators and barriers (e.g., built environment, parents' security perception for AT of children). |
| Fernández et al. (2019) | *AT*- 48.5% of children aged 6–10 years commuted active (walk) during the previous week.  *AT-* 24.3% of children aged 6–10 years commuted active (bike) during the previous week. | 27.1% for boys  21.4% for girls  12.9% for boys  11.4% for girls | First bachelor's theses on PA-related indicators of the GM initiative. Cross-sectional study indexed in the official repository of the University of the Republic and performed with 70 children of a private Primary school from Rivera city in 2019. Relevant national research group (GIARH) focused on PA and health. / Participants were only from one Uruguayan city and the study sample was small. The available data were insufficient to report a grade. / Influence of AT-related factors (e.g., socioeconomic status, seasons of the year). |
| National Public Education Administration (2020) | *SCH* - 72.9% of children from Primary school attended PE lessons.  *SCH* - 52.4% of the total number of schools are considered 'active schools'. | N/A | Official document containing descriptive data about the number of students and professors of PE course for primary levels in 2020. The National Secretary of Sport has calculated the proportion of students covered by the specialists. / Lack of public information on schools developing active school policies and description of their approach. / Disaggregated data by gender. |
| National Public Education Administration (2014) | *SCH* - at least two weekly PE lessons of 40 minutes for the Primary school. | N/A | Official document created by the specific committee for primary levels reporting that PE is mandatory in Formal Education. Description of the main approaches (e.g., teaching profile) and characteristics (e.g., time) to PE classes. / Lack of public information on participation in PE classes according to specific children's requirements (e.g., physical fitness card) or infrastructure features (e.g., indoor PA space). / Information of schools with PA opportunities in addition to PE classes. Influence of children's characteristics (e.g., functional disabilities, residential zone) for participation in PE classes. |
| National Public Education Administration (2020) | *SCH* - at least two hours per week of PE lessons for the Secondary school. | N/A | Official document created by the specific committee for secondary levels reporting that PE is mandatory in formal education. Description of the main approaches (e.g., teaching profile) and characteristics (e.g., time) to PE classes. / Lack of public information on participation in PE classes according to specific youth’s requirements (e.g., physical fitness card) or infrastructure features (e.g., indoor PA space). / Information of high schools with PA opportunities in addition to PE classes. Influence of youth's characteristics (e.g., functional disabilities, residential zone) for participation in PE classes. |
| National Secretary of Sport (2020) | *GOV-* a total of 30 programmes and projects of the Uruguayan sports system were identified.  *GOV-* a total of 63 Leading Sport Entities were identified across the country. | N/A | Uruguay's National Sport Plan 2015–2020 describes and evaluates the practice of sport and PA in terms of its development at community, federative and educational levels. Specifically, summarises the policies, programs and projects carried out by the National Secretariat of Sport. / Limited information on policies, programs, funding, and actions specifically for children and adolescents. / Disaggregate information on policies, programmes, funding, and actions according to childhood and adolescence. |
| National Secretary of Sport (2019) | *GOV-* a total of 63 Leading Sport Entities were identified across the country.  *SP-* 16% of children and adolescents aged 6–19 years are enrolled in a federated sport. | N/A  27.0% for boys  4.6% for girls | Cross-sectional national survey conducted by the National Observatory of Sport in 2019 among the Leading Sport Entities (n=63) across the country. In 2015, this survey was conducted for the first time among the country's Leading Sports Entities. / Of the total Entities, 15 submitted incomplete or no data. No follow-up data. The age range included participants over 17 years old. / Data according to SP other than federated sports and disaggregate information by childhood and adolescence. |
| National Secretary of Sport / National Public Education Administration (2018) | *PF*- children and adolescents aged 10–14 years with a mean of 132.1cm in the SLJ test (muscle power)  *PF*- children and adolescents aged 10–14 years with a mean of 23.2cm in the Sargent test (muscle power)  *PF*- children and adolescents aged 10–14 years with a mean of -2.6cm in the Toe Touch test (flexibility) | 138.5cm for boys  123.8cm for girls  24.1cm for boys  22.4cm for girls  -4.3cm for boys  -0.9cm for girls | Cross-sectional nationally representative evaluation-program in 2017 that includes 6755 participants enrolled in 5th and 6th grade of Primary school. A total of 133 schools took part in the evaluation process (103 public and 30 private). The program included registration of anthropometric measures and fitness field-test (i.e., standing long jump, vertical jump, flexibility, and speed/agility). / The dataset is not available, and the technical report was inappropriate to report a grade. / Appropriate data on PF components in childhood and adolescence, specifically for cardiorespiratory fitness. |
| National Youth Institute (2020) | *COM*- 42.4% of adolescents and adults aged 12–35 years do sports in public facilities. | 45.5% for boys  37.8% for girls | Nationally representative cross-sectional survey conducted periodically since 2008. The 4th National Survey on Adolescence and Youth was performed in 2018–2019 including 6543 participants. / Age range includes adult population. No follow-up data. / Information on CE specifically for childhood and adolescence. Data on the use of public infrastructure according to indicators. Systematized information on public spaces throughout the country, both those that depend on the National or Departmental Governments. Analyse factors related to the use of public spaces, in terms of youths (e.g., characteristics, security perceived), infrastructure (e.g., location, accessibility), or public activities provided by PA professionals (e.g., type, targets). |
| Prates et al. (2021) | *SP*- 60.0% of children aged 6–12 years participated in organized PA programs.  *SP*- 20.5% of children aged 6–12 years participated in sports competitions. | N/A  30.0% for boys  10.5% for girls | First bachelor's theses on PA-related indicators of the GM initiative. Cross-sectional study indexed in the official repository of the University of the Republic and performed with 55 children of a private Primary school from Rivera city in 2020. National research group (GIARH) focused on PA and health. / Participants were only from one Uruguayan city and the study sample was small. The available data were insufficient to report a grade. / Influence of SP-related factors (e.g., socioeconomic status, type of sport). |
| Uruguayan Parliament (2007, 2008, 2011, 2019, 2020) | *GOV*- 49.5% of influence on youth physical activity (following a standardized method to grade the GOV indicator ^b^ ). | N/A | At the national level, 7 laws were identified containing information about governmental influence on PA opportunities, however, only 5 referred specifically to childhood and adolescence. These laws involve the National Secretary of Sport, National Administration of Public Education, Ministry of Public Health, Ministry of Education and Culture, Honorary Commission for the Prevention, Control, and Eradication of Violence in Sport, Commission of Sport Projects, Ministry of Economy and Finances, and Congress of Mayors. Four (26,28–30) out of 5 laws defined the organisations that lead its development. In 2 laws (26,29) there are recognised structures to create reports. / None of the identified laws included information about the budget for its development. / Accessible information on public funds and resources for the implementation of PA promotion strategies specifically for children and youth. Link the different PA-related indicators initiatives in childhood and adolescence with the laws concerning this purpose. |
| World Health Organization (2019) | *AT*- 50.5% of adolescents aged 13–17 commuted active to/from school at least 4 days per week.  *PA*- 13.8% of adolescents aged 13–17 years meet the PA international guidelines ^a^.  *SB*- 37.1% of adolescents aged 13–17 years reported less than 3 hours per day of screentime. | 53.1% for boys  48.6% for girls | Nationally representative cross-sectional survey conducted periodically since 2008 with the WHO technical support. The Uruguay GSHS was carried out by the Ministry of Public Health and performed in 2019 with a participation of 3162 students. Sixty-six schools from all the cities were selected at the first stage and all the students were eligible to participate at the second stage. / Data not disaggregated by capital city and the rest of the country compared to the GSHS 2012. / Surveillance data on PA-related indicators according to important demographic (e.g., country's regions) or socioeconomical (e.g., parents' education level) factors. |
|  |  | 18.8% for boys  9.5% for girls  35.7% for boys  38.2% for girls |  |
|  |  |  |  |

^a^ 60 minutes per day during the previous 7 days. ^b^ The sequence applied was as follows: Number and Breadth of Relevant Policies; Identified supporting actions; Identified accountable organisation; Identifiable reporting structures; Identified funding; and Monitoring and evaluation plan. In: Ward MR, Tyler R, Edwards LC, Miller MC, Williams S, Stratton G. The AHK-wales report card 2018: policy measures - is it possible to ‘score’ qualitative data? *Health Promot Int.* (2021) 36:1151–9. doi: 10.1093/heapro/daaa118

Abbreviations: AP, Active play; AT, Active transportation; COM, Community and built environment; CIET, 'Research Centre for the Tobacco Epidemic'; CUiiDARTE, 'University Center for Research, Innovation and Arterial Diagnostics'; FAM, Family and Peers; GIARH, 'Human Performance Analysis Research Group'; GM, Global Matrix; GSHS, Global School-based Student Health Survey; GOV, Government; N/A, not applicable; PA, physical activity; PE, Physical Education; PF, Physical fitness; SB, Sedentary behaviour; SCH, School environment; SLJ, standing long jump test; SP, organised sport and physical activity participation; WHO, World Health Organization.
